# Does Promotion Orientation Help Explain Why Future-Orientated People Exercise and Eat Healthy?

**DOI:** 10.3389/fpsyg.2017.01202

**Published:** 2017-07-25

**Authors:** Taciano L. Milfont, Roosevelt Vilar, Rafaella C. R. Araujo, Robert Stanley

**Affiliations:** ^1^Centre for Applied Cross-Cultural Research and School of Psychology, Victoria University of Wellington Wellington, New Zealand; ^2^School of Psychology, Massey University Auckland, New Zealand

**Keywords:** eating behavior, health knowledge, attitudes, practice, future orientation, regulatory focus, replication, cross-cultural research

## Abstract

A study with United States undergraduate students showed individuals high in concern with future consequences engage in exercise and healthy eating because they adopt a promotion orientation, which represents the extent to which individuals are inclined to pursue positive gains. The present article reports a cross-cultural replication of the mediation findings with undergraduate samples from Brazil and New Zealand. Promotion orientation mediated the association between concern with future consequences and exercise attitudes in both countries, but the associations for healthy eating were not replicated—which could be explained by distinct obesity prevalence and eating habits in these socio-cultural contexts. We discuss theoretical and practical implications of the findings for promoting health behavior.

## Introduction

Health-related behaviors can entail negative short-term consequences, including the loss of pleasurable experiences, expenditure of time and money, and physical and psychological discomfort associated with physical exertion. At the same time, such behaviors can yield significant long-term benefits and gains, involving improved physical fitness and enhanced general health and well-being (e.g., Ouellette et al., 2005; Joireman et al., 2012). A decision to exercise and eat healthy thus involves making an intertemporal trade-off by accruing present costs in order to achieve delayed rewards. It is thus likely that the importance individuals attach to the future consequences of their behavior may influence their willingness to engage in health-related behaviors (see, e.g., Hall and Fong, 2007; Adams and Nettle, 2009; Gellert et al., 2012).

Indeed, several studies have shown that individuals who are more future orientated are more likely to engage in healthy behavior, while less likely to engage in risk-taking behavior, compared to those who are more present oriented. To illustrate, future-oriented individuals are more likely to use a condom (Appleby et al., 2005), accept free sunscreen (Orbell and Kyriakaki, 2008), manage aggression while driving (Moore and Dahlen, 2008), exercise (Adams and Nettle, 2009), eat healthy (Piko and Brassai, 2009), and have more regular sleep schedules (Peters et al., 2005). Moreover, future orientation has been shown to predict health and well-being indictors over time (Chua et al., 2015).

What can explain the link between future orientation and health-related behavior? Research has suggested that regulatory focus—the extent to which individuals are inclined to pursue positive gains or avoid negative losses (Higgins, 1997)—is a mechanism by which future considerations translate into healthy behaviors (e.g., Joireman et al., 2012). Regulatory focus theory proposes two main strategies for goal attainment (Higgins, 1997; Higgins et al., 2001). A *promotion* orientation is concerned with the achievement of *ideal* self-goals (e.g., hope, wishes, and aspirations) and involves the eager pursuit of gains and successes. A *prevention* orientation entails striving to attain *ought* self-goals (e.g., duties, obligations, and responsibilities) and includes strategies aimed at vigilantly avoiding losses and failures.

That regulatory focus may help to explain the link between future orientation and health-related behavior is consistent with studies showing associations between temporal perspective and regulatory focus (or other analogous constructs). For example, Ouellette et al. (2005) found that when individuals who scored high on a future orientation measure were made to consider ideal possible selves they tended to increase their exercise behavior. These findings suggest that greater temporal distance might facilitate the pursuit of promotion goals, and therefore suggest that future orientation and regulatory focus are theoretically related constructs (see also Pennington and Roese, 2003; Mogilner et al., 2008).

Two studies have explicitly examined the associations between future and promotion/prevention orientations. In a sample of employees from a company in Netherlands (*N* = 85), Zacher and de Lange (2011) found that future time orientation was positively correlated with a promotion orientation, whereas present time orientation was positively correlated with prevention orientation. Joireman et al. (2012) investigated the associations between considerations of temporal consequences, regulatory focus, and health-related outcomes. They report two studies with United States undergraduate students showing that consideration of *future* consequences was positively correlated with promotion orientation, as well as with exercise attitudes and intentions (Study 1; *N* = 119) and healthy eating attitudes and intentions (Study 2; *N* = 232). In addition, promotion orientation (but not prevention orientation) was significantly related to exercise and healthy eating attitudes and intentions.

Notably, Joireman et al. (2012) sought to test whether promotion orientation explains why those high in consideration of *future* consequences are more likely to exercise and eat healthy among their sample of United States undergraduate students. Path analyses confirmed the proposed mediation model. In Study 1, the authors found that consideration of *future* consequences predicted exercise attitudes via promotion orientation, and exercise attitudes mediated the prediction of promotion orientation on exercise intentions. Similarly, in Study 2 consideration of *future* consequences predicted healthy eating attitudes via promotion orientation, and healthy eating attitudes mediated the prediction of promotion orientation on healthy eating intentions. Visually, their proposed and confirmed mediation model was as follows: CFC-Future → Promotion → Attitudes → Intention.

The goal of the present study was to provide a cross-cultural replication of the Joireman et al. (2012) findings in samples of undergraduate students from Brazil and New Zealand. There is evidence indicating that culture plays an important role in shaping individuals’ time perspective and regulatory focus (e.g., Kurman and Hui, 2011; Sircova et al., 2014) and health behavior (e.g., Blodgett et al., 2015; Higgs and Thomas, 2016), and health indicators also vary across countries, which is illustrated by the Obesity Atlas^1^. However, we do not make predictions concerning possible cross-cultural differences; instead we expect to replicate the Joireman et al. (2012) findings across our samples. Although selected based on convenience, Brazil and New Zealand differ in many dimensions of cultural variability (Milfont, 2009) and differ when compared to the United States (e.g., Inglehart and Baker, 2000). Replicating the mediation model in distinct socio-cultural contexts will provide evidence for the role of both temporal considerations and regulatory focus in influencing health attitudes and behavioral intentions. The study goal aligns with recent calls for more replication and systematic accumulation of knowledge in psychological science (Open Science Collaboration, 2015; Munafò et al., 2017).

Specifically, we predicted that individuals who place greater emphasis on considerations of *future* consequences of their behavior would be more likely to indicate in self-report measures more positive attitudes and intentions toward exercise and healthy eating. Importantly, we predicted that the mediation model proposed and confirmed by Joireman et al. (2012): promotion orientation would mediate the link between consideration of *future* consequences and exercise and healthy eating attitudes and intentions.

## Materials and Methods

### Participants and Procedure

We analyzed psychology student data collected as part of broader survey questionnaire conducted in Brazil (*N* = 136, 70.6% female, *M*_age_ = 21.5, *SD*_age_ = 6.3) and New Zealand (*N* = 144, 70.8% female, *M*_age_ = 19.1, *SD*_age_ = 1.3). The survey study was approved by the School of Psychology Human Ethics Committee under delegated authority of Victoria University of Wellington’s Human Ethics Committee. New Zealand participants completed an online survey for partial course credit, and the Brazilian students completed a paper survey without rewards. All measures were translated into Brazilian-Portuguese using a bilingual committee approach. Although comparable regarding gender distribution, the New Zealand sample was younger, *t*(177.5) = 4.16, *p* < 0.001, *d* = 0.53. We included sex and age as covariates in the mediation path analysis by allowing them to correlate with all variables in the models.

### Measures

#### Temporal Orientation

Participants completed the new version of the consideration of future consequences scale (CFC-14) described by Joireman et al. (2012). The CFC-14 distinguishes factors assessing concern for *future* consequences (CFC-Future: “I am willing to sacrifice my immediate happiness or wellbeing in order to achieve future outcomes”) and concern for *immediate* consequences (CFC-Immediate: “My convenience is a big factor in the decisions I make or the actions I take”). Items were rated on a scale from 1 (*very uncharacteristic of me*) to 7 (*very characteristic of me*). The two-factor model fitted the combined data well (CFI = 0.917, RMSEA = 0.059, SRMR = 0.065), and a multi-group analysis confirmed metric (ΔRMSEA = 0.001) and partial scalar equivalence (ΔRMSEA = 0.002; freeing item 2 intercept) of the two-factor CFC-14 model in Brazil and New Zealand.

#### Regulatory Focus

Participants completed the 18-item measure of regulatory focus (Lockwood et al., 2002), which consists of two subscales designed to measure promotion (“I frequently imagine how I will achieve my hopes and aspirations”) and prevention orientations (“In general, I am focussed on preventing negative events in my life”). Items were rated on a scale from 1 (*not at all true of me*) to 7 (*very true of me*). The two-factor model provided acceptable fit to the combined data (CFI = 0.848, RMSEA = 0.074, SRMR = 0.083), and a multi-group analysis confirmed metric (ΔRMSEA = 0.001) and partial scalar equivalence (ΔRMSEA = 0.003; freeing item 13 intercept).

#### Attitudes and Behavioral Intention

Three items assessed exercise attitudes (Joireman et al., 2012): (a) regular exercise is essential to good health, (b) regular physical activity makes one feel better, and (c) I enjoy physical exercise. Three items assessed healthy eating attitudes: (a) eating healthy is essential to my well-being, (b) I enjoy eating healthy, and (c) I feel great personal satisfaction when I eat healthy. Attitude items were rated on a scale from 1 (*strongly disagree*) to 7 (*strongly agree*). Future exercise intentions were assessed with a single item: “Next week how many times do you plan to exercise (how many different exercise sessions)?” Healthy eating intentions was assessed by asking participants to think about future breakfasts, lunches, and dinners and to rate how healthy those meals would be (1 = *not healthy*; 10 = *very healthy*). To test the cross-cultural equivalence of the health measures, we considered a parsimonious two-factor model with exercise and healthy eating attitudes. This two-factor model fitted the data well (CFI = 0.986, RMSEA = 0.059, SRMR = 0.021) and a multi-group analysis confirmed metric (ΔRMSEA = 0.013) and scalar equivalence (ΔRMSEA = 0.003) in Brazil and New Zealand.

## Results

**Table 1** presents correlations and descriptive statistics. As noted above, confirmatory factor analysis on the CFC-14 scale confirmed the two-factor model in both countries, providing further support for the two-factor conceptualization of CFC (see also Supplementary Material). We first ran mediation path analysis for each country separately. The fit of the single-country models to the data was satisfactory for both countries (CFI > 0.90, SRMR < 0.080).

**Table 1:** Correlations and descriptive statistics.

	α	*M*	*SD*	1	2	3	4	5	6	7	8	α	*M*	*SD*
1. CFC-Future	0.81	4.63	0.90	1	-0.12	0.53^∗∗∗^	0.45^∗∗∗^	0.11	0.07	0.09	-0.03	0.74	5.06	0.90
2. CFC-Immediate	0.75	3.93	0.86	-0.31^∗∗∗^	1	0.11	0.27^∗∗^	-0.10	-0.07	-0.22	-0.20	0.69	2.85	0.75
3. Promotion	0.83	5.08	0.82	0.48^∗∗∗^	-0.11	1	0.49^∗∗∗^	0.14	-0.03	0.00	0.04	0.84	7.15	1.17
4. Prevention	0.72	4.47	0.90	0.16	-0.11	0.14	1	-0.00	-0.01	-0.03	-0.04	0.81	6.00	1.45
5. Exercise attitudes	0.82	5.72	1.12	0.33^∗∗∗^	-0.01	0.37^∗∗∗^	0.02	1	0.30^∗∗∗^	0.54^∗∗∗^	0.19^∗^	0.64	4.27	0.64
6. Exercise intentions	–	3.56	3.05	0.24^∗∗^	0.00	0.24^∗∗^	-0.11	0.45^∗∗∗^	1	0.31^∗∗∗^	0.26^∗∗^	–	2.19	2.05
7. Healthy eating attitudes	0.87	5.36	1.24	0.38^∗∗∗^	-0.15	0.31^∗∗^	0.08	75^∗∗∗^	0.42^∗∗∗^	1	0.39^∗∗∗^	0.74	3.86	0.86
8. Healthy eating intentions	–	6.81	1.80	0.23^∗∗^	-0.16^∗^	0.19^∗^	-0.06	0.43^∗∗∗^	0.39^∗∗∗^	0.58^∗∗∗^	1	–	7.21	1.54


*Correlations below diagonal for New Zealand sample (N = 144), and above diagonal for Brazil sample (N = 136). CFC-Future, consideration of future consequences; CFC-Immediate, consideration of immediate consequences. ^∗^p < 0.05, ^∗∗^p < 0.01, ^∗∗∗^p < 0.001 (two-tailed)*.

**FIGURE 1:**
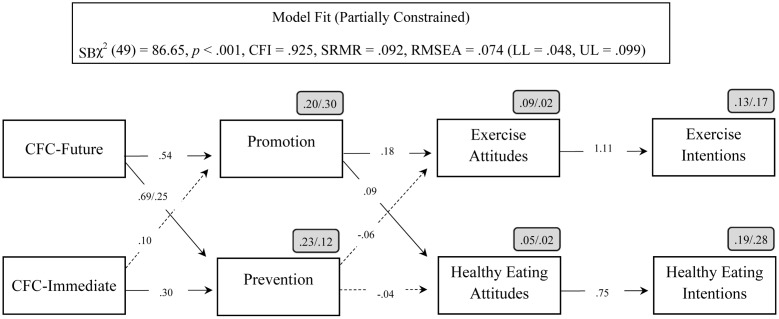
Partially invariant multi-group path model linking CFC subscales with health outcomes via regulatory focus orientation. Coefficients shown are unstandardized paths. Values separated by forward slash are for Brazil and New Zealand, respectively. For simplicity, the correlations between the constructs as well as the covariance of both sex and age in all constructs are not shown in the figure. CFC-Future, consideration of *future* consequences; CFC-Immediate, consideration of *immediate* consequences; CFI, comparative fit index; SB, Sattora-Bentler; SRMR, standardized root mean square residual; RMSEA, root mean square error approximation; LL, lower limit; UL, upper limit.

We then ran a multi-group mediation model in which all regression paths were free to vary across groups (group-specific model), followed by a model in which all the regression paths were fixed to be equal across groups (universal model). The group-specific model [χ^2^(28) = 48.71, *p* = 0.009, CFI = 0.959, SRMR = 0.045, RMSEA = 0.073 (LL = 0.036, UL = 0.106)] showed a significantly better fit [Δχ^2^(22) = 47.82; *p* < 0.01] than the universal model [χ^2^(50) = 96.53, *p* < 0.001, CFI = 0.907, SRMR = 0.105, RMSEA = 0.082 (LL = 0.057, UL = 0.106]. Allowing the relation between CFC-Future and prevention orientation to vary freely across groups resulted in a partially invariant universal model with acceptable fit [χ^2^(49) = 86.65, *p* < 0.001, CFI = 0.925, SRMR = 0.092, RMSEA = 0.074 (LL = 0.048, UL = 0.099)], and comparable to the fully variant group-specific model [Δχ^2^(21) = 37.94; *p* = 0.013]. **Figure 1** presents the final multi-group mediation model.

After achieving the final constrained model cross countries, we then examined the particular mediation paths. As can be seen in **Table 2**, our findings replicate those reported by Joireman et al. (2012) regarding exercise attitudes and intention. In both countries, CFC-future predicts exercise intentions via promotion orientation (albeit marginally in Brazil), which in turn predicts exercise intentions via attitudes. At the same time, we did not replicate their findings for healthy eating attitudes. Only the mediation of attitudes on the promotion–intention link replicated in New Zealand.

**Table 2:** Summary of indirect effects tests.

Indirect effect tested	Path A		Path B		Path C’		Indirect effect (AB)		
	(X → M)		(M → Y._X_)		(X → Y._M_)		95% confidence interval		
	β	*p*	β	*p*	β	*p*	Lower	Point	Upper
**Brazil**									
Exercise									
CFC-Future → Promotion → Attitudes	0.547	0.000	0.180	0.036	0.052	0.520	-0.008	0.099^†^	0.213
Promotion → Attitudes → Intentions	0.204	0.030	0.314	0.000	-0.092	0.332	0.011	0.064^∗^	0.147
Healthy Eating									
CFC-Future → Promotion → Attitudes	0.547	0.000	-0.012	0.915	0.083	0.483	-0.120	-0.007	0.124
Promotion → Attitudes → Intentions	0.028	0.786	0.351	0.000	0.070	0.423	-0.061	0.010	0.088
**New Zealand**									
Exercise									
CFC-Future → Promotion → Attitudes	0.485	0.000	0.287	0.004	0.238	0.038	0.050	0.173^∗^	0.369
Promotion → Attitudes → Intentions	0.383	0.000	0.427	0.000	0.093	0.373	0.102	0.164^∗∗∗^	0.243
Healthy Eating									
CFC-Future → Promotion → Attitudes	0.485	0.000	0.173	0.105	0.281	0.024	-0.015	0.084	0.218
Promotion → Attitudes → Intentions	0.308	0.000	0.575	0.000	0.019	0.802	0.090	0.176^∗∗∗^	0.276

^†^*p = 0.07, ^∗^p < 0.05, ^∗∗∗^p < 0.001, Path A, relationship between independent variable (IV) and mediator; Path B, relationship between mediator and dependent variable (DV), controlling for IV; Path C’, direct effect of IV on DV, controlling for mediator. Lower, lower bound of confidence interval; Point, point estimate; Upper, upper bound of confidence interval. Indirect effect is significant if confidence interval does not include zero. CFC, consideration of future consequences*.

## Discussion

The present study provides a cross-cultural replication of the findings reported by Joireman et al. (2012) examining whether regulatory focus mediates the relationship between consideration of temporal consequences and exercise/healthy eating attitudes and intentions. Consistent with their findings, promotion orientation mediated the association between CFC-Future and exercise attitudes in samples from Brazil and New Zealand. Individuals who are more aware of, and attach greater weight to, the potential *future* consequences of their behavior are more likely to pursue positive gains and adopt ideal self-goals (i.e., hopes, dreams, and aspirations), which in turn makes them more likely to uphold more positive attitudes toward physical exercise.

The findings have theoretical and practical importance. Our results provide further evidence for the intrinsic association between future time perspective and regulatory focus, and between these constructs and health behavior (e.g., Hall and Fong, 2007). The results also suggest that individuals’ future thinking can shield self-control failure, perhaps due to greater ability to delay gratification (Watson and Milfont, 2017). In line with the argument offered by Joireman et al. (2012), interventions aimed at increasing exercise attitudes and intentions should focus on encouraging individuals to consider the future consequences of their actions, and there is empirical evidence showing that such interventions can work (Hall and Fong, 2003).

However, we did not replicate their mediation findings regarding healthy eating attitudes. Alternative explanations can be proposed for this finding, but here we focus on socio-cultural contexts as a boundary condition. Culture help shape individuals’ time perspective and regulatory focus (e.g., Kurman and Hui, 2011; Sircova et al., 2014), and attitudes and behaviors related to eating and physical exercise (Blodgett et al., 2015; Higgs and Thomas, 2016). We expected that the mediation role of promotion orientation on the association between consideration of future consequences and health outcomes proposed by Joireman et al. (2012) would be culturally invariant; an expectation overall supported by the results. However, our findings also show that the mediation model might not hold for all outcomes and in all socio-cultural contexts. Joireman et al. (2012) considered university students from the United States, a country with a higher prevalence of fast food chains and high-fat diet. A comparison of the obesity prevalence among adults in these countries shows that the United States comes first (34.5%), followed by New Zealand (30.7%) and Brazil (14.7%)^2^. We speculate that a context of high levels of obesity coupled with government initiatives would enhance the salience of healthy eating to individuals, resulting in stronger associations between future and promotion orientations with attitudes and intentions related to healthy eating observed in the United States and New Zealand but not in Brazil. Further replication studies in distinct socio-cultural contexts are needed to confirm this possibility.

Moreover, we relied on self-report measures of behavioral intentions, and future research might employ behavioral measures of both exercise and diet. This is important because intentions might be more susceptible to change or to be provisional due to the temporal interval between them and the behavior (Sutton, 1998). Future research testing the mediation model with longitudinal data would also strengthen confidence in our findings.

Overall, our findings shed light on the role of temporal orientation in the context of health behavior by indicating that greater concern for future consequences might encourage exercise (and to some degree, healthy eating) through a specific type of self-regulatory system, one that represents goals, aspirations and accomplishments. Our findings together with those from previous results (e.g., Ouellette et al., 2005; Orbell and Kyriakaki, 2008; Joireman et al., 2012) may have important implications in the health domain, suggesting that strategies designed to increase health behavior should emphasize the future benefits of these behaviors.

## Author Contributions

TM designed the study and coordinated the project. RV and RA run the statistical analysis. RS helped with data collection. All authors contributed to writing the manuscript.

## Conflict of Interest Statement

The authors declare that the research was conducted in the absence of any commercial or financial relationships that could be construed as a potential conflict of interest.

## Footnotes

1 http://www.worldobesity.org/data/
