# Supplementary material for: Does Promotion Orientation Help Explain Why Future-Orientated People Exercise and Eat Healthy?
Source: Front Psychol. 2017 Jul 25;8:1202. doi: 10.3389/fpsyg.2017.01202 (PMC5524921; doi:10.3389/fpsyg.2017.01202)
Supplement: Supplementary file 1 [file Data_Sheet_1.docx]

**Supplementary Material**

Milfont TL, Vilar R, Araujo RCR and Stanley R (2017). Does Promotion Orientation Help Explain Why Future-Orientated People Exercise and Eat Healthy? Front. Psychol. 8:1202. doi: 10.3389/fpsyg.2017.01202

Joireman and colleagues (2012, Study 2) run confirmatory factor analysis and showed that the two-factor model underlying the CFC-14 had good fit to the data [SBχ2(69) = 97.69, p = .013, GFI = .943, CFI = .965, RMSEA = .043 (LL = .020, UL = .062)], and that CFC-Future and CFC-Immediate were not strongly correlated (Φ = −.37). However, it is important to note that they allowed seven correlated errors (including two across factors) to achieve this fit.

We report the results of the confirmatory factor analysis testing the two-factor CFC-14 model separately in Brazil and New Zealand (see Figures S1 and S2). The fit is reasonable in New Zealand without any correlated errors, and the factors are not strongly correlated (Φ = −.44). The fit in Brazil was poorer, even after allowing two correlated errors, and again the factors are not strongly correlated (Φ = −.37). Further studies should examine the factor structure of the CFC-14 in Brazil, paying special attention to items 2 and 5 which showed low factor loadings. Indeed, the model in Brazil showed better fit when these two items were deleted and the two correlated errors were kept: SBχ2 (52) = 70.61, p = .043, CFI = .936, SRMR = .055, RMSEA = .051 (LL = .009, UL = .079).

CFC1

CFC2

CFC6

CFC7

CFC8

CFC13

CFC14

CFC3

CFC4

CFC5

CFC9

CFC10

CFC11

CFC12

**Brazil sample (*N* = 136)** – Model Fit:

SBχ2 (74) = 117.28, *p* = .001, CFI = .872, SRMR = .082, RMSEA = .042, LL = .048, UL = .087

.52

.16

.56

.67

.52

.48

.66

.55

.54

.03

.47

.67

.76

.22

.51

.31

-.37

**Figure S1.** Confirmatory factor analysis of the CFC-14 Scale in the Brazil sample

*Note*. CFC, consideration of future consequences; SB, Sattora-Bentler; CFI, comparative fit index; SRMR, standardized root mean square residual; RMSEA, root mean square error approximation; LL, lower limit; UL, upper limit. All paths significant, *p* < .05.

CFC1

CFC2

CFC6

CFC7

CFC8

CFC13

CFC14

CFC3

CFC4

CFC5

CFC9

CFC10

CFC11

CFC12

.67

.69

.47

.60

.50

.66

.70

.63

.55

.34

.66

.51

.75

.35

-.44

**New Zealand sample (*N* = 144)** – Model Fit:

SBχ2 (74) = 110.06, *p* = .004, CFI = .914, SRMR = .076, RMSEA = .058, LL = .033, UL = .080

**Figure S2.** Confirmatory factor analysis of the CFC-14 Scale in the New Zealand sample

*Note*. CFC, consideration of future consequences; SB, Sattora-Bentler; CFI, comparative fit index; SRMR, standardized root mean square residual; RMSEA, root mean square error approximation; LL, lower limit; UL, upper limit. All paths significant, *p* < .05.

**Table S1**. Correlations and Descriptive Statistics

|  | α | *M* | *SD* | 1 | 2 | 3 | 4 | 5 | 6 | 7 | 8 | α | *M* | *SD* |
| --- | --- | --- | --- | --- | --- | --- | --- | --- | --- | --- | --- | --- | --- | --- |
| 1. CFC-Future | .81 | 4.63 | .90 | 1 | -.12 | .53*** | .45*** | .11 | .07 | .09 | -.03 | .74 | 5.06 | .90 |
| 2. CFC-Immediate | .75 | 3.93 | .86 | -.31*** | 1 | .11 | .27** | -.10 | -.07 | -.22 | -.20 | .69 | 2.85 | .75 |
| 3. Promotion | .83 | 5.08 | .82 | .48*** | -.11 | 1 | .49*** | .14 | -.03 | .00 | .04 | .84 | 7.15 | 1.17 |
| 4. Prevention | .72 | 4.47 | .90 | .16 | .11 | .14 | 1 | -.00 | -.01 | -.03 | -.04 | .81 | 6.00 | 1.45 |
| 5. Exercise attitudes | .82 | 5.72 | 1.12 | .33*** | -.01 | .37*** | .02 | 1 | .30*** | .54*** | .19* | .64 | 4.27 | .64 |
| 6. Exercise intentions | - | 3.56 | 3.05 | .24** | .00 | .24** | -.11 | .45*** | 1 | .31*** | .26** | - | 2.19 | 2.05 |
| 7. Healthy eating attitudes | .87 | 5.36 | 1.24 | .38*** | -.15 | .31** | .08 | 75*** | .42*** | 1 | .39*** | .74 | 3.86 | .86 |
| 8. Healthy eating intentions | - | 6.81 | 1.80 | .23** | -.16* | .19* | -.06 | .43*** | .39*** | .58*** | 1 | - | 7.21 | 1.54 |

*Note.* Correlations below diagonal for New Zealand sample (*N* = 144), and above diagonal for Brazil sample (*N* = 136). CFC-Future = Consideration of *future* consequences. CFC-Immediate = Consideration of *immediate* consequences. **p* < .05. ***p* < .01. ****p* < .001 (two-tailed).

**Table S2.** Summary of Indirect Effects Tests

| Indirect effect tested | Path A | | Path B | | Path C' | | Indirect effect (AB) | | |
| --- | --- | --- | --- | --- | --- | --- | --- | --- | --- |
|  | **(X → M)** | | **(M → Y._X_)** | | **(X → Y._M_)** | | **95% confidence interval** | | |
|  | **β** | ***p*** | **β** | ***p*** | **β** | ***p*** | **Lower** | **Point** | **Upper** |
| Brazil |  |  |  |  |  |  |  |  |  |
| Exercise  CFC-Future → Promotion → Attitudes | .547 | .000 | .180 | .036 | .052 | .520 | -.008 | .099† | .213 |
| Promotion → Attitudes → Intentions | .204 | .030 | .314 | .000 | -.092 | .332 | .011 | .064* | .147 |
| Healthy Eating  CFC-Future → Promotion → Attitudes | .547 | .000 | -.012 | .915 | .083 | .483 | -.120 | -.007 | .124 |
| Promotion → Attitudes → Intentions | .028 | .786 | .351 | .000 | .070 | .423 | -.061 | .010 | .088 |
| New Zealand |  |  |  |  |  |  |  |  |  |
| Exercise  CFC-Future → Promotion → Attitudes | .485 | .000 | .287 | .004 | .238 | .038 | .050 | .173* | .369 |
| Promotion → Attitudes → Intentions  Healthy Eating | .383 | .000 | .427 | .000 | .093 | .373 | .102 | .164*** | .243 |
| CFC-Future → Promotion → Attitudes | .485 | .000 | .173 | .105 | .281 | .024 | -.015 | .084 | .218 |
| Promotion → Attitudes → Intentions | .308 | .000 | .575 | .000 | .019 | .802 | .090 | .176*** | .276 |

Note: † *p* = .07, **p < .*05*, *** p <* .001, Path A = relationship between independent variable (IV) and mediator; Path B = relationship between mediator and dependent variable (DV), controlling for IV. Path C’ = direct effect of IV on DV, controlling for mediator. Lower = lower bound of confidence interval; Point = point estimate; Upper = upper bound of confidence interval. Indirect effect is significant if confidence interval does not include zero. CFC = Consideration of Future Consequences.

**Measurement Invariance Test**

As noted in the main document, we also test for measurement invariance for the scales used across Brazil and New Zealand. We provide the specific results below.

**CFC SCALE**

MODEL FIT

CFA TOTAL SAMPLE: CFI = .917, TLI = .901, RMSEA = .059, SRMR = .065

CFA NEW ZEALAND: CFI = .905, TLI = .886, RMSEA = .060, SRMR = .065

CFA BRAZIL: CFI = .777, TLI = .733, RMSEA = .086, SRMR = .089

INVARIANCE

CONFIG: CFI = .848, TLI = .818, RMSEA = .074, SRMR = .083

METRIC : CFI = .829, TLI = .810, RMSEA = .075, SRMR = .104

DELTAS CFI .019, RMSEA .001

SCALAR: CFI = .756, TLI = .748, RMSEA = .087, SRMR = .113

DELTAS CFI .073, RMSEA .012

PARTIAL SCALAR: CFI = .806, TLI = .799, RMSEA = .077, SRMR = .111

DELTAS CFI .023, RMSEA .002 (Intercept of Item 2 was freed)

**RFQ SCALE**

MODEL FIT

CFA TOTAL SAMPLE: CFI = .858, TLI = .838, RMSEA = .085, SRMR = .080

CFA NEW ZEALAND: CFI = .745, TLI = .709, RMSEA = .087, SRMR = .116

CFA BRAZIL: CFI = .735, TLI = .697, RMSEA = .103, SRMR = .094

INVARIANCE

CONFIG: CFI = .740, TLI = .703, RMSEA = .095, SRMR = .106

METRIC: CFI = .731, TLI = .710, RMSEA = .094, SRMR = .119

DELTAS CFI .009, RMSEA .001

SCALAR: CFI = .672, TLI = .665, RMSEA = .101, SRMR = .140

DELTAS CFI .059, RMSEA .007

PARTIAL SCALAR: CFI = .694, TLI = .687, RMSEA = .097, SRMR = .136

DELTAS CFI .037, RMSEA .003 (Intercept of Item 13 was freed)

**EXERCISE AND EATING ATTITUDES SCALE**

MODEL FIT

CFA TOTAL SAMPLE: CFI = .986, TLI = .973, RMSEA = .059, SRMR = .021

CFA NEW ZEALAND: CFI = .999, TLI = .997, RMSEA = .017, SRMR = .024

CFA BRAZIL: CFI = .952, TLI = .909, RMSEA = .083, SRMR = .049

INVARIANCE

CONFIG: CFI = .989, TLI = .970, RMSEA = .053, SRMR = .038

METRIC: CFI = .969, TLI = .954, RMSEA = .066, SRMR = .089

DELTAS CFI .020, RMSEA .013

SCALAR: CFI = .966, TLI = .957, RMSEA = .063, SRMR = .113

DELTAS CFI .003, RMSEA .003
